# P-1623. The Burden of COVID-19: A Longitudinal Study of SARS-CoV-2 Immunity and Infection Risks

**DOI:** 10.1093/ofid/ofaf695.1799

**Published:** 2026-01-11

**Authors:** Kathleen M Lindsey, Theresa Kowalski-Dobson, Carmen Gherasim, Anna Buswinka, Ashley Eckard, Zijin Chu, Rebecca Tutino, Gabriel Simjanovski, David Manthei, Emily Stoneman, Riccardo Valdez, Aubree Gordon

**Affiliations:** University of Michigan School of Public Health, Ann Arbor, MI; University of Michigan School of Public Health, Ann Arbor, MI; Michigan Medicine, University of Michigan, Ann Arbor, Michigan; University of Michigan School of Public Health, Ann Arbor, MI; University of Michigan School of Public Health, Ann Arbor, MI; University of Michigan School of Public Health, Ann Arbor, MI; University of Michigan School of Public Health, Ann Arbor, MI; University of Michigan School of Public Health, Ann Arbor, MI; University of Michigan School of Public Health, Ann Arbor, MI; Michigan Medicine, University of Michigan, Ann Arbor, Michigan; Michigan Medicine, University of Michigan, Ann Arbor, Michigan; University of Michigan, Ann arbor, MI

## Abstract

**Background:**

Understanding the burden of SARS-CoV-2 throughout the pandemic is essential for characterizing the virus’s epidemiology and evaluating public health interventions. Here, we describe the Immunity Associated with SARS-CoV-2 (IASO) cohort and assess the risk of COVID-19 across the pandemic to better understand long-term immunity from past exposures.

**Methods:**

IASO is a prospective cohort established in October of 2020. Participants were surveyed at enrollment, weekly for symptoms, bi-monthly for social distancing and vaccination updates, and following known exposures. Serum samples were collected bi-monthly, and acute infection testing was available throughout the study. Infections were identified through both acute testing and serological monitoring. Case counts were age-standardized to the population structure of the State of Michigan to evaluate differences in incidence. We estimated infection rates using Poisson regression with and without spline functions for time. Risk was compared by exposure level using Cox proportional hazards models.

**Results:**

In total, 3,357 adults contributed over 5,900 person-years of observation. Participants experienced 1,781 symptomatic infections, yielding an incidence of 33.5 cases per 100 person-years (95% CI: 32.2-34.8) over the study period. During the peak of COVID-19 incidence in the 2022 Omicron wave, the rate of symptomatic infection reached 158.9 cases per 100 person-years (95% CI: 126.8-196.8). Compared to unvaccinated individuals, vaccinated participants had a 60% lower risk of symptomatic infection (HR = 0.40, 95% CI 0.30-0.54) within the first year of enrollment. Among vaccinated participants with one prior infection, each additional vaccine dose was associated with a 41% reduction in reinfection risk (HR = 0.59 , 95%CI 0.48-0.73) within a year of initial infection.

**Conclusion:**

Vaccination conferred protection during each wave of the pandemic, with vaccinated individuals consistently at lower risk of symptomatic infection than those unvaccinated. Additionally, participants with a prior infection who remained up to date with vaccination continued to benefit from added protection. Taken together, these findings support adherence to vaccination recommendations regardless of prior infection status.

**Disclosures:**

Aubree Gordon, Ph.D., M.P.H., Janssen Pharmaceuticals: Advisor/Consultant|Sanofi Pasteur: Advisor/Consultant

